# Cost Analysis of Various Low Pathogenic Avian Influenza Surveillance Systems in the Dutch Egg Layer Sector

**DOI:** 10.1371/journal.pone.0033930

**Published:** 2012-04-16

**Authors:** Niels Rutten, José L. Gonzales, Armin R. W. Elbers, Annet G. J. Velthuis

**Affiliations:** 1 Business Economics, Wageningen University, Wageningen, The Netherlands; 2 Department of Epidemiology, Crisis Organization and Diagnostics, Central Veterinary Institute (CVI), Wageningen, The Netherlands; 3 Department of Farm Animal Health, Faculty of Veterinary Medicine, Utrecht University, Utrecht, The Netherlands; University of New South Wales, Australia

## Abstract

**Background:**

As low pathogenic avian influenza viruses can mutate into high pathogenic viruses the Dutch poultry sector implemented a surveillance system for low pathogenic avian influenza (LPAI) based on blood samples. It has been suggested that egg yolk samples could be sampled instead of blood samples to survey egg layer farms. To support future decision making about AI surveillance economic criteria are important. Therefore a cost analysis is performed on systems that use either blood or eggs as sampled material.

**Methodology/Principal Findings:**

The effectiveness of surveillance using egg or blood samples was evaluated using scenario tree models. Then an economic model was developed that calculates the total costs for eight surveillance systems that have equal effectiveness. The model considers costs for sampling, sample preparation, sample transport, testing, communication of test results and for the confirmation test on false positive results. The surveillance systems varied in sampled material (eggs or blood), sampling location (farm or packing station) and location of sample preparation (laboratory or packing station). It is shown that a hypothetical system in which eggs are sampled at the packing station and samples prepared in a laboratory had the lowest total costs (i.e. € 273,393) a year. Compared to this a hypothetical system in which eggs are sampled at the farm and samples prepared at a laboratory, and the currently implemented system in which blood is sampled at the farm and samples prepared at a laboratory have 6% and 39% higher costs respectively.

**Conclusions/Significance:**

This study shows that surveillance for avian influenza on egg yolk samples can be done at lower costs than surveillance based on blood samples. The model can be used in future comparison of surveillance systems for different pathogens and hazards.

## Introduction

Low Pathogenic Avian Influenza (LPAI) viruses are commonly found in aquatic wild birds, which are assumed to be the main reservoir [1], [2], [3], for transmission to commercial poultry [4]. In poultry, LPAI viruses typically cause mild respiratory problems or decrease in egg production and/or water and feed intake which cause economic damage to the poultry sector [5]. However, the main concern about LPAI-viruses of H5- and H7-subtypes is that they can mutate to a High Pathogenic Avian Influenza (HPAI) virus. This has happened before, e.g. in the USA [6], Mexico [7], Italy [8], Chile [9], Netherlands [10], and Canada [11]. An outbreak of HPAI would cause a serious threat to both the poultry sector and public health. In poultry, large epidemics with major economic consequences have been reported [1], [12]. Both LPAI and HPAI viruses can infect humans and have the potential to cause an outbreak of influenza in the human population [13], [14]. To date in a total of 553 cases humans were infected with the Asian HPAI H5N1 virus strain have been reported worldwide out of which 323 were fatal [15]

Because of the animal and public health risks and economic impact, prevention of an AI outbreak of H5- or H7-subtype is a priority for the European Union (EU). Therefore, a serological surveillance system for poultry has been implemented in the member states (MS) of the EU. The aim of this surveillance program is detection of infection with LPAI H5- and H7-subtypes and contribute to the demonstration of freedom of H5- and H7-subtypes [16]. The EU requires a surveillance system for LPAI H5- and H7-subtypes which is stratified throughout the territory of the whole MS, so that samples can be considered representative for the whole MS [16]. Depending on the total number of poultry farms in a MS a minimal number of poultry farms is required to be sampled and tested, with a minimum of five blood samples per poultry house and ten per farm [16]. For the Netherlands the minimal requirement of the EU is that 60 broiler farms, 60 egg layer farms, 60 turkey farms and 60 meat duck farms are sampled per year [16]. However, next to the EU surveillance system, the Dutch poultry industry implemented a more intensive AI surveillance system for the whole poultry sector [17]. This is because the risk of a fast spreading epidemic is considered to be high as most poultry farms are concentrated in a few areas of the country [18].

This study focuses on alternative surveillance systems for egg layer farms. The egg layer sector is the most targeted poultry sector for surveillance in the EU and the Netherlands [19]. In the Dutch LPAI surveillance system, all egg layer farms with indoor-housed hens are required to send in 30 blood samples of randomly selected hens once a year [20]. By contrast, free-range and organic laying hen farms are required to send in 30 blood samples of randomly selected hens every 90 days [20]. This is because outdoor ranged poultry is considered more likely to have contact with wild birds and face a higher risk of an AI-introduction than indoor housed poultry [4].

Test validation studies, suggest that the accuracy of diagnostic tests using egg samples is comparable to that of tests using blood samples [21], [22]. Furthermore, egg samples have been used previously for the detection of infected farms during an outbreak of LPAI viruses of H7N2 subtype [23]. This observation suggests that using egg samples for surveillance might be equally effective as using blood samples. The advantage of the egg surveillance is that the sampling of eggs is flexible. In addition, egg sampling is more desirable from the perspective of animal welfare as hens do not have to be distressed and sampled in an invasive manner. On the other hand, egg-sampling has disadvantages at the laboratory level. Preparing egg-yolk samples is technically more complex and more time consuming, hence it might be that processing yolk samples is more expensive than the processing of blood samples. Most advantages and disadvantages can be reflected in monetary terms, which enhances decision making [24].

Efficiency of a surveillance program can be measured in economic terms. This will not only include epidemiologic effectiveness of disease detection but also costs of this detection, the economic benefit from preventing an epidemic or the additional costs of missing an outbreak and subsequent problems. Therefore, with an economic analysis it is possible to choose a strategy that has the least costs and thereby rationally allocate scarce resources [25]. For instance, for Bluetongue and Bovine tuberculosis economic analysis has been used to identify the most profitable strategy of controlling or monitoring a disease [26], [27].

For policy makers it is necessary to have an economic comparison of different surveillance systems. This study will present the differences in costs between surveillance systems that originate from the before-mentioned advantages and disadvantages of using eggs as a sample commodity. Because hypothetical alternative surveillance systems should have a probability of detecting LPAI virus introductions comparable to the current system based on blood samples (i.e. an equal benefit), a cost minimization analysis is used, where the system with the lowest costs is preferred [28]. Thus, the objective of this study is to perform a cost minimization analysis of various LPAI surveillance systems for Dutch egg layer farms based either on blood or egg sampling with equal probability of detecting a LPAI virus introduction.

## Materials and Methods

### The Dutch egg layer sector

The Dutch poultry sector consists of 838 indoor farms and 270 outdoor ranging farms producing a combined number of 9.6 billion eggs [29]. The product boards for Livestock Meat and Eggs does not register owners of less than 250 laying hens as farmers as they are considered hobby “farmers”. The Dutch egg layer farms were mainly concentrated in the south-eastern and central part of the country. The eggs of egg layer farms were distributed every two or three days to 86 packing stations where they are sorted into size categories and then distributed to retailers and industry. There were 19 large packing stations that process more than 100 million eggs per year and 67 small packing stations [29]. The small packing stations were often a farm that sorts and sells his own eggs. A few small egg laying farmers are known to sell their own eggs unsorted directly to the consumer (e.g. on a local market). These are ignored in this study as this number was negligible.

### Alternative surveillance systems

The various LPAI surveillance systems evaluated are summarized in Table 1. They can differ in five aspects: i) sample material: blood and/or eggs, ii) location of sampling: egg layer farm and/or packing station, iii) sampling frequency, iv) number of samples obtained per farm and v) location of sample preparation: central laboratory and/or packing station.

**Table 1:** Overview of the evaluated Low Pathogenic Avian Influenza surveillance systems.

Surveillance		Sampling												Sample preparation					
system		Material				Location				Frequency/yr		Number		Location				Method	
		Blood		Eggs		Layer		Packing		Indoor	Outdoor	Indoor	Outdoor	Central		Packing		Robot	Hand
						farm		station		farms	farms	farms	farms	lab		station			
B/F/L	1	X				X				1	4	30	30	X				X	
E/F/L	2			X		X				1	4	35	35	X				X	
E/P/L	2			X				X		1	4	35	35	X				X	
E/P/P	2			X				X		1	4	35	35			X		X	
E/P/LP	2			X				X		1	4	35	35	X	a	X	b	X	
E/FP/LP	2			X		X	c	X	d	1	4	35	35	X	a	X	b	X	
BE/FP1/L	2	X	e	X	f	X		X		1	4	30	35	X				X	
BE/FP2/LP	2	X	e	X	f	X	c	X	d	1	4	30	35	X	a	X	b	X	

1 Current system, implemented in practice.

2 Hypothetical alternative.

a Small packing stations send in eggs.

b Large packing station have a robot.

c Farms that deliver to small packing stations send in eggs.

d Large packing stations have a robot.

e Blood samples for conventional farms.

f Egg samples for outdoor ranging farms.

Surveillance system acronyms: The first part is the sampled material either blood (B), eggs (E) or a combination of blood and eggs (BE). The second part is the sampling location either farm (F), packing station (P), a combination of farm and packing station (FP), blood sampled at the farm and eggs sampled at the packing station (FP1) or blood sampled at the farm and eggs sampled and prepared at the packing station (FP2). The third part is the location of sample preparation either laboratory (L), packing station (P) or a combination of laboratory and packing station (LP).

Acronyms are used to describe the different systems. The first part represents the sample material: Blood or Eggs. The second part represents the sampling location: Farm for egg layer farm or Packing station. The third part represents the location where the sample is prepared: Lab for central laboratory or Packing station. For example, the current surveillance system is represented by Blood/Farm/Lab meaning that blood samples are taken at the farm and send to a central laboratory for preparation and testing.

### Effectiveness of surveillance systems

The effectiveness (sensitivity) of the surveillance (here referred to as Surveillance Sensitivity (*SSe*)) carried out in egg layer farms in the Netherlands using blood samples or egg samples was estimated by using a scenario tree model [30]. First, the flock sensitivity was estimated (*F_se_* = probability of detection at the farm level) using the sample sizes mentioned in Table S1, the sensitivity of the ELISA test using blood or egg samples and a design prevalence of 10%. The design prevalence represents a hypothetical minimum prevalence expected should a LPAI infection be present in the flock. Using this *F_se_* the *SSe* was estimated which consisted of two components: 1) surveillance in indoor layer farms and 2) surveillance in outdoor layer farms. The design prevalence used to estimate the *SSe* was 0.05% [19]. The estimated *SSe* is equal for all evaluated surveillance systems, because all systems sample the same number of farms with the same frequency. Note that for this evaluation a perfect specificity of the surveillance systems is assumed. This is due to the fact that any seropositive detection has to be followed up until false positive results are excluded [16]. A detailed description of the scenario tree model is provided as Appendix S1.

### Sampling

**Figure 1:**
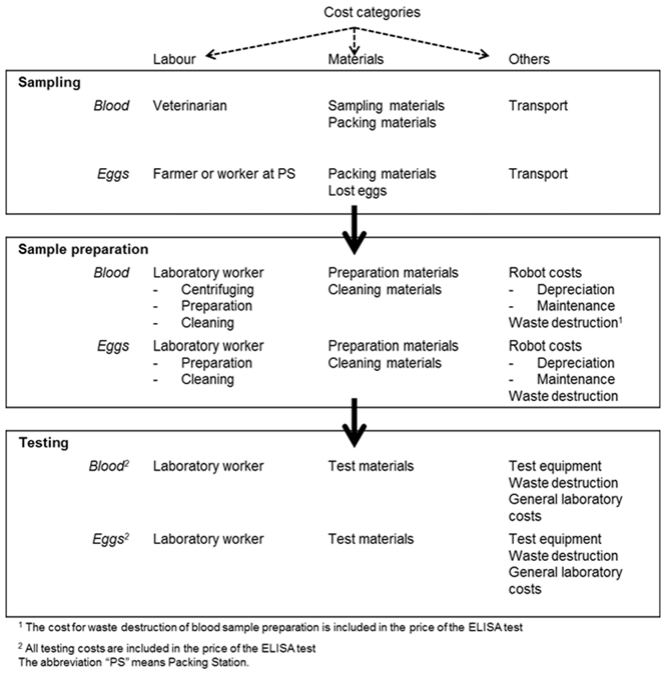
Flow chart of the process of sampling for blood and eggs in the various surveillance systems.

Figure 1 shows a flow chart with the different processes of sampling for the current system using blood samples and hypothetical alternatives using egg samples. Blood samples for AI surveillance are taken by a licenced veterinarian (as required by the Dutch authorities) at the egg layer farms. This sampling is mostly done in combination with the compulsory sampling for Newcastle Disease (ND) and *Mycoplasma gallisepticum* (MG) surveillance, that requires farms to sample at the latest nine weeks before the slaughter date [31]. Eggs can be collected at either egg layer farms and/or packing stations depending on the surveillance system. At the farm, every day each hen produces one egg [32]. The eggs of all chickens will automatically be removed from laying nests and collected in trays containing thirty eggs each day. A tray of eggs can be considered a random sample as eggs of various laying nests are mixed in this process, It is assumed that the egg collection can be performed by the farmer and/or a worker of the packing station as eggs are easy to collect (by randomly taking one tray and five eggs from another tray) and to trace by the official printed date and unique farm code on the egg. The number of egg samples is higher than the number of blood samples to correct for the lower egg production due to a possible LPAI infection [33].

### Sample preparation

Blood samples are centrifuged at a central laboratory just after arrival to separate serum from other blood components. Next a sample is taken of the serum by a robot from the vial. This sample is transferred to an ELISA-plate and diluted to a concentration appropriate for testing.

The sample preparation of eggs would be different from that of blood. Trays of eggs can be handled by a specialised robot: a needle guided by a robot penetrates the egg-shell, the egg white and finally the egg-yolk. Then a yolk sample is taken and transferred to an ELISA-plate and diluted to a concentration appropriate for testing [34]. A sample preparation robot is considered in this study because sampling egg-yolk by hand is very labour intensive and labour costs are high in the Netherlands [34].

### Testing

Next, the sample is tested using an automated ELISA procedure. Samples are tested for the presence of antibodies against AI viruses using a commercial ELISA test kit which classifies samples as positive or negative. Studies evaluating the performance of the ELISA test using egg-yolk and blood samples showed that (once infected animals seroconvert) the sensitivity and specificity for both blood and egg yolk samples are very similar [21], [22], [35]. Jeong et al. [22] estimated sensitivities of 100% and specificities 91.8% and 90.9% for blood and yolk samples respectively.

### Cost calculations

The total costs of surveillance system 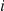 (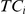) include costs related to the following activities: sampling (i.e. 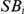 for blood and 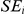 for eggs), sample preparation (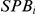 for blood and 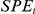 for eggs), testing (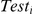), waste processing (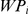), transport to the central laboratory (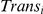), communication to the farmer (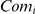) and confirmation testing for positive results (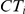):
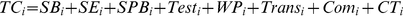
(1)The cost calculations of each activity will be explained in detail in the following paragraphs and the inputs in Table S1.

The costs related to blood sampling (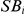) includes the call out charge of the veterinarian (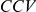), labour cost for preparation and sampling (i.e. the amount of hours multiplied with the veterinary tariff: (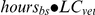), the cost of used materials (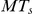) and the cost of packing material on the farm (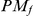):
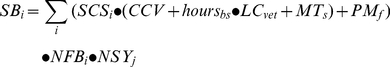
(2)Here 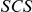 is the share of the cost for AI surveillance. In addition, 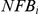 is the number of farms that is sampled by blood sampling which depends on the surveillance program. And 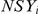 is the number of samplings per year which depends on the type of farm (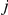), i.e. indoor farms or outdoor farms.

The cost related to egg sampling (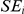) includes the labour cost for sampling eggs from individual farms and the cost of packaging on the sampling location (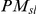) in which 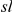
is either packing station or farm:
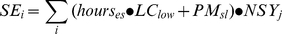
(3)Here 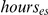 is the time needed per sampling, 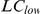 the labour cost of a worker at the packing station or at the farm and 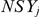 is the number of samplings per year per farm.

The cost relating to sample preparation for blood (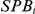) includes cost for spinning down sampled blood, cost for used materials (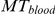) and cost for the sample preparation robot (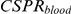).
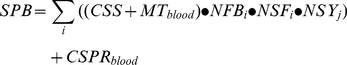
(4)Here 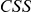 is the cost per sample for spinning down the blood and booking in the samples when arrived at the laboratory, 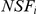 is the number of samples per flock.

The cost for the sample preparation robot (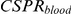) was calculated using the following equation:
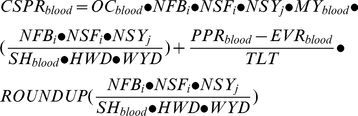
(5)Here, 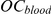 is the labour cost per year of analysts who operate the robot and 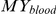 the maintenance cost per year. The number of robots needed per year is calculated by dividing the number of samples prepared per year (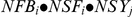) by the robot capacity. Here 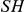 is the number of samples per hour, 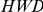 the number of working hours per day and 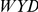 the number of working days per year. Then the yearly cost of the investment of one robot is calculated by dividing the difference between the purchase price (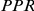) and the end value of the robot (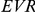) by the technical life time (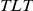). For multiple robots the investment costs should be multiplied by the number of whole robots needed to prepare all samples.

The cost related to sample preparation of eggs (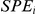) includes cost for the sample preparation robot (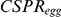), booking the samples when arrived at the laboratory (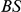) and cost for used materials (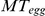).
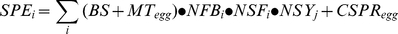
(6)The cost for the sample preparation robot (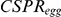) was calculated using a similar equation as for blood:
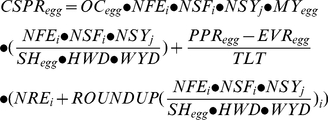
(7)Here (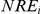) represents the number of robots for egg samples as some surveillance systems contain a fixed number of robots (namely on all or some packing stations) instead of the number of robots required for the number of samples to be processed.

The cost related to the transport of sampled material and/or prepared samples to the central laboratory (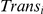) either cost for sending the material with the postal service or transport by a courier:
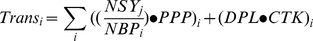
(8)Here (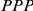) represents the price of sending a package with the postal service. From the number of samplings per year (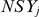) and the number of sampling batches per package (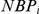) the number of packages per year is calculated. And (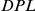) is the distance in kilometres between packing station and central laboratory is multiplied by (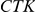) the cost of transport per kilometre.

The cost related to the ELISA test (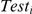) includes the price of one test (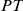) and the number of tests performed per year.
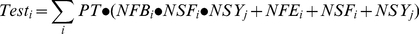
(9)The cost related to waste processing (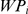) includes the difference in the amount of waste produced when samples are prepared from one egg (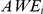) and the amount of waste produced when samples are prepared from one blood sample (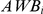) multiplied by the number of egg samples and the price for incinerating the waste (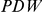).
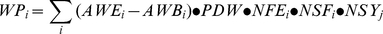
(10)For blood samples the cost for waste processing are included in the test price as this is a commercial price that includes all laboratory costs. The use of eggs for sample preparation is known to give more waste material than the use of blood samples therefore the extra waste processing costs are calculated.

The cost made to communicate the test results to the farmers (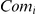) includes the price of sending a notification (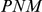) and the number of samplings per year (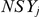):
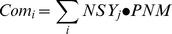
(9)The cost of the confirmation tests performed due to positive results (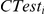) is (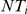) the number of tests per year (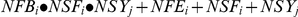) multiplied by the expected percentage of positive results (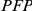) and the price of the confirmation test (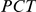) summarized with the cost of transporting samples to the Dutch national reference laboratory (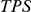).
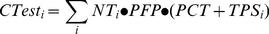
(10)For the confirmation test the originally tested samples are send to the Dutch national reference laboratory and retested in a confirmation test. 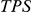 consist of the transport distance for a central lab to the Dutch national reference laboratory times the transport cost per kilometre. The cost of additional tests and measures after a true positive result is obtained by the surveillance system are considered to be cost of an AI outbreak or epidemic and are therefore not included.

### Inputs

The inputs summarized in Table S1, were used for the calculation of cost of the various surveillance systems. These inputs were obtained from official reports, scientific literature and experts. One expert was a professional poultry veterinarian, the second one was a sector specialist of the product board for poultry, meat and eggs and the last one was a veterinarian working in the central laboratory who has developed the sample preparation robot for eggs.

### Sensitivity analysis

A sensitivity analysis was performed to assess and identify the inputs that influence the total costs the most. Each individual input was changed with +10% and −10% and the total costs were calculated. This analysis was carried out using the add-in software TopRank 5.5 for Excel of Palisade Decision Tools [36].

## Results

### Effectiveness of surveillance

The Flock sensitivity (F*se*) sampling 30 blood or 35 egg samples was estimated to be 95.5% (95% Confidence Intervals (CI): 95.2–96.0) and 94.2% (95% CI: 93.0–95.0) respectively. The sensitivity of surveillance (*SSe*) was estimated to be 96.5% (95%CI: 96.4–96.6) using blood samples and 96.4% (95%CI: 96.3–96.5) using egg samples. Thus, it was concluded that the effectiveness of surveillance is the same regardless of the type of sample used.

### Total costs of the surveillance systems

**Figure 2:**
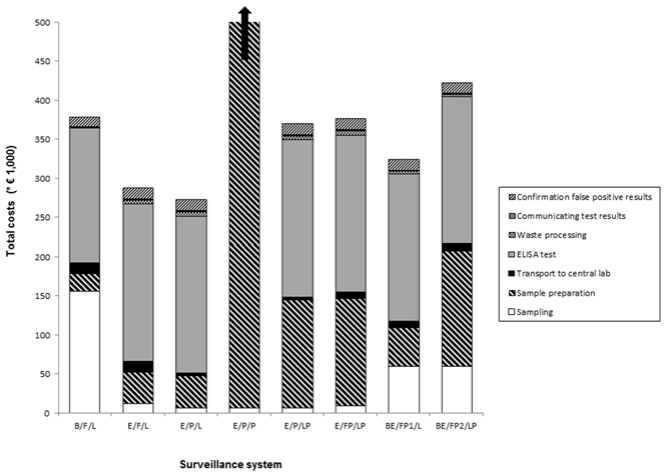
Total net costs of various surveillance systems and differentiation to various activities. Surveillance system acronyms: The first part is the sampled material either blood (B), eggs (E) or a combination of blood and eggs (BE). The second part is the sampling location either farm (F), packing station (P), a combination of farm and packing station (FP), blood sampled at the farm and eggs sampled at the packing station (FP1) or blood sampled at the farm and eggs sampled and prepared at the packing station (FP2). The third part is the location of sample preparation either laboratory (L), packing station (P) or a combination of laboratory and packing station (LP).

Figure 2 and Table 2 show that the Eggs/Packing station/Packing station system had total costs of € 2,354,734 and was thereby the most expensive.

**Table 2:** Total costs of LPAI surveillance systems.

	*Costs for each activity and total costs of various surveillance systems*							
Activity within surveillance system	B/F/L	E/F/L	E/P/L	E/P/P	E/P/LP	E/FP/LP	BE/FP1/L	BE/FP2/L
Sampling	157,609	13,628	8,116	8,116	8,116	10,286	61,118	61,118
Sample preparation	22,524	40,358	40,358	2,111,808	137,838	137,838	49,766	147,246
Transport to central lab	13,053	13,053	3,263	13,053	2,945	6,800	7,506	8,467
ELISA test	172,398	201,131	201,131	201,131	201,131	201,131	188,679	188,679
Waste processing	-	5,415	5,415	5,415	5,415	5,415	3,068	3,068
Communicating test results	967	967	967	967	967	967	967	967
Confirmation false positive results	12,124	14,144	14,144	14,144	14,144	14,144	13,269	13,269
**Total Costs**	378,674	288,695	273,393	2,354,632	370,554	376,580	324,372	422,813

The total costs are the sum of the costs for surveillance on indoor and outdoor farms.

Surveillance system acronyms: The first part is the sampled material either blood (B), eggs (E) or a combination of blood and eggs (BE). The second part is the sampling location either farm (F), packing station (P), a combination of farm and packing station (FP), blood sampled at the farm and eggs sampled at the packing station (FP1) or blood sampled at the farm and eggs sampled and prepared at the packing station (FP2). The third part is the location of sample preparation either laboratory (L), packing station (P) or a combination of laboratory and packing station (LP).

The lowest costs € 273,494 were estimated for the Eggs/Packing station/Lab system (Table 2). This was a result of the lower costs for sampling which was partially offset by higher costs of sample preparation. Compared to the Eggs/Packing station/Lab system the other systems were more costly: plus 39% for Blood/Farm/Lab, plus 6% for Eggs/Farm/Lab, plus 761% for Eggs/Packing station/Packing station, plus 36% for Eggs/Packing station/Lab+Packing station, plus 38% for Eggs/Farm+Packing station/Lab+Packing station, plus 24% for Blood+Eggs/Farm+Packing station(1)/Lab and plus 60% for Blood+Eggs/Farm+Packing station(2)/Lab.

### Costs of different activities

Table 2 shows that sampling costs of system Blood/Farm/Lab was 11–20 times higher than the costs of systems Eggs/Farm/Lab and Eggs/Packing station/Lab. By contrast, the costs for sample preparation of systems Eggs/Farm/Lab and Eggs/Packing station/Lab were about twice the sampling costs of Blood/Farm/Lab. The rates charged by the veterinarian for blood sampling caused the high costs for sampling in the Blood/Farm/Lab system. The higher purchase price and lower capacity of a robot for egg-preparation caused the high costs for sample preparation in the Eggs/Farm/Lab and Egg/Packing station/Lab systems. For Blood/Farm/Lab, testing costs were lower than in the other systems, which was caused by the lower sample sizes needed in the Blood/Farm/Lab system (30 blood samples per farm per sampling) compared to the other systems (35 egg samples per farm per sampling). The costs for transport, waste processing, communication, transport of positive results and the confirmation test were of minor importance as they are only 5% of the total costs of a surveillance system.

Table 3 shows that in each surveillance system the costs for surveillance per outdoor farm were higher than for surveillance per indoor farm. This difference was caused by the higher sampling frequency required for outdoor farms. Costs for sampling eggs at farm level were much lower than blood sampling. The costs on a packing station was higher than other systems when preparation is located on packing stations. Costs per packing station were € 2,237 higher when only large packing station are preparing samples (Table 3).Preparing blood samples at the central laboratory resulted in much lower costs € 22,524 than preparing egg samples € 40,358. The costs were the highest € 49,766 when both blood and egg samples were prepared in a central laboratory (Table 3).

**Table 3:** Costs per firm (i.e. farm, packing station or laboratory) per year.

	Sampling		Sample preparation		
	Blood	Egg	Blood	Eggs	Blood+Eggs
Farm (€/farm/year)					
Indoor	67.45	7.05	nr	nr	nr
Outdoor	374.41	28.60	nr	nr	nr
Packing station (€/packing station/year)					
Sampling	nr	94.37	nr	nr	nr
Sampling+sample preparation	nr	94.37	nr	24,556	nr
Small	nr	38.97	nr	nr	nr
Large	nr	1,230	nr	26,793	nr
Laboratory (€/laboratory/year)					
Laboratory	nr	nr	22,524	40,358	49,766

### Sensitivity analysis

**Figure 3:**
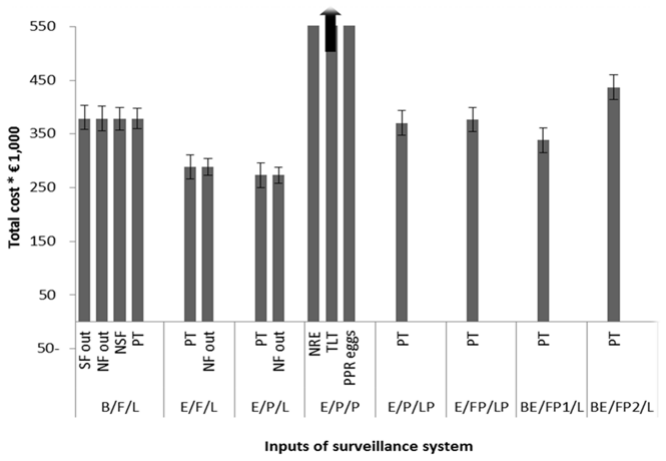
Sensitivity analysis for the most influential inputs (below the bars). For each surveillance system only inputs are shown that cause at least a 5% increase and decrease of total costs. Surveillance system acronyms: The first part is the sampled material either blood (B), eggs (E) or a combination of blood and eggs (BE). The second part is the sampling location either farm (F), packing station (P), a combination of farm and packing station (FP), blood sampled at the farm and eggs sampled at the packing station (FP1) or blood sampled at the farm and eggs sampled and prepared at the packing station (FP2). The third part is the location of sample preparation either laboratory (L), packing station (P) or a combination of laboratory and packing station (LP).Other abbreviations: SF out: Sampling Frequency outdoor farms, NF out: Number of outdoor farms, NSF: Number of Samples per Farm, PT: Price of one Test, NRE: Number of Robot for Eggs, TLT: Technical lifetime of a robot and PPR eggs: Purchase Price Robot for eggs.

Figure 3 shows the results for the sensitivity analysis, the effect on total costs are shown for individual inputs that change the total costs by more than 5%. Some individual inputs in the model had a substantial influence on the total costs of the Blood/Farm/Lab system: A 10% increase in the following inputs resulted in an increase in total costs of: 6.6% for sampling frequency for outdoor farms, 6.0% for number of outdoor farms, 5.6% for samples per flock for outdoor farms and 5.2% for price per test. For example when the number of outdoor farms was increased by 10% (i.e. 270+27) the output (total costs) increased by 6.0%. Inputs associated with the robot were the most influential inputs in the Eggs/Packing station/Packing station system. A 10% increase in the following inputs resulted in an increase in total costs of: 8.9% for number of robots, 8.5% for technical lifetime and 7.7% for robot price. Test price and number of outdoor farms were the most influential inputs for systems Eggs/Farm/Lab (7.9% and 5.6%) and Eggs/Packing station/Lab (8.3% and 5.4%) The test price, was the most influential input in the other systems. Here the change in total costs ranged from 5.2% to 6.7%.

## Discussion

The objective of this study was to perform a cost analysis of various LPAI surveillance systems for Dutch egg layer farms based either on blood or egg sampling. System Eggs/Packing station/Packing station was expected to have the highest total costs as it was designed with a sample preparation robot for eggs on each single packing stations. It can be concluded that the systems Eggs/Farm/Lab, Eggs/Packing station/Lab, Eggs/Packing station/Lab+Packing station, Eggs/Farm+Packing station/Lab+Packing station and Blood+Eggs/Farm+Packing station(1)/Lab had lower total costs than the current Blood/Farm/Lab system. The difference in total costs between the Blood/Farm/Lab system and the Eggs/Farm/Lab and Eggs/Packing station/Lab systems were −€ 89,965 and −€ 105,267 respectively. For policy makers matters of animal welfare and vulnerability to fraud will also be an issue. The Eggs/Packing station/Lab system seems interesting in this perspective because packing stations are independent of farmers and all eggs are printed with a unique identification number when they arrive at a packing station. The sampling frequency and the sample size were the most influential inputs of the economic model. The reason for their importance was that these inputs determine the amount of work in every activity of a surveillance system. It was expected that the egg surveillance systems would be cheaper than the Blood/Farm/Lab system. The main reason was that sampling costs for eggs are lower. This is explained by the difference in labour costs: a veterinarian is more expensive than a farmer. Although the costs related to the sample preparation robot were relatively high they did not exceed the savings in the sampling activities. Sampling eggs at a packing station seemed more efficient and therefore total cost for Eggs/Packing station/Lab were lower than other systems based on egg samples.

In this study a cost analysis was used to compare systems with equal epidemiological effectiveness. The study was limited as it did not show the relation between cost and effectiveness. So cost and benefits of the actual detection of a LPAI virus remain unknown although this could be relevant information.

If the hypothetical systems would be implemented in practice, the implementation and start-up would result in additional cost. In this study calculations of costs were done for the hypothetical situation in which all systems would be implemented and working. The implementation and start-up costs are however incidental and therefore beyond the scope of the current research. For all egg layer farms it was assumed that they supply eggs to a packing station. However it is known that some farms sell unsorted eggs directly to the end users. When the packing station is the sampling location this type of farms should be sampled separately. As the number of those farms is small only a limited increase of total costs can be expected. For the testing it was assumed that the confirmation test has no false negative results. Only after a virus is isolated on a farm measures will be taken. So considering the small amount of false negative results and the knowledge that no measures are taken when no virus is isolated, this assumption could in the perspective of total costs of surveillance result in a small underestimation of total costs.

In western countries labour costs are generally high and therefore the use of robots in a laboratory would result in lower costs. In countries with lower labour costs conventional sample preparation could be the cheapest and most simple solution. Lower labour costs will probably decrease the total costs of a surveillance system. Apart from costs, reliability should be also considered. A robot is expected to be more consistent than manual sample preparation because it will prepare samples for testing at a constant quality and can be controlled better, which may increase the reliability of the tests performed in a laboratory.

The preparation of samples on a packing station resulted in inefficient use of the robot and therefore to high total costs. An egg-based system reduces the cost for sampling whereas it increased the cost for sample preparation compared to a blood-based system. For the number of samples that are taken in one year 3 robots would have sufficient capacity. The Eggs/Packing station/Packing station system was highly inefficient because it used 29 times the minimal number of robots. For instance a packing station that only processes the eggs of one indoor farm will use the robot for sample preparation ¼ of a day a year.

The most influential inputs were of a chosen value based on the epidemiological effectiveness of the surveillance systems or the current situation. Therefore, the values are given based on epidemiological data (e.g. sampling frequency for indoor farms) or facts (e.g. the number of indoor farms). For decision makers it is however important to know that the test price was an important factor in the total costs of a surveillance system. In recent years the number of outdoor farms increased in the Dutch egg layer sector [37]. It seems reasonable to assume that this trend will continue in the coming years [37] and thus increase total costs of any AI surveillance system.

The rate veterinarians charge for sampling blood was an important factor in the costs of the blood surveillance system. The costs were only attributed for one third to LPAI surveillance and two third to the ND and Mg surveillance, as blood samples are taken for three surveillance systems. Therefore, the costs for the veterinarian should be considered as sunk costs (i.e. costs that are incurred and cannot be recovered) in case of a combined surveillance. Meaning that the surveillance costs for ND and Mg will increase because the same veterinary costs are attributed to two diseases (only to ND and Mg) instead of three (not to LPAI anymore).

In practice, farmers usually combine the blood sampling for AI with the pre-slaughter test for ND and MG. In the calculations it was assumed that this combined test occurs every year. In practice however it occurs that conventional egg laying farmers start-up a new flock during year 1 and slaughter them in year 3 so no sampling is done in year 2 for ND and MG, some farmers might also skip the sampling for AI [31]. Furthermore, total costs for surveillance may be underestimated because non-combined samplings can occur in practice.

When blood is sampled from laying hens the catching and handling of the birds will cause stress in the flock. Therefore, a drop in egg-production could be expected and subsequently additional indirect costs to the surveillance system. Quantification of this drop in production has not been shown in literature. In practise this effect seems to be limited [38]. The costs caused by a loss of production are neglected in our calculations. A reduction of stress for the birds or less indirect costs for farmers might be arguments in favour of using egg sampling for surveillance.

A study similar to the study in this paper has been done on the costs of surveillance for bovine tuberculosis. In that study it was concluded that sampling bulk-tank milk for surveillance will require a higher sampling frequency to have the same effectiveness as other testing methods [39]. However, using bulk-milk samples results in lower costs than other sampling methods, including blood testing [26]. For the egg layer sector a surveillance system based on egg-sampling has been suggested for *Salmonella enterica* [*40*] *.* However Thomas et al. [40] did not provide any comparison to other testing methods nor is there an economic analysis of such a system available. These previous findings indicate that the use of egg yolk samples can be interesting for surveillance on other diseases in poultry and for more intensive surveillance.

Combining the surveillance on LPAI described in this paper with surveillance programs based on egg samples for other diseases (e.g. the salmonella surveillance) would most probably reduce the combined costs for these surveillance systems.

In this paper economies of scale and inflation or discounting have not been considered. Hypothetical alternative systems would have more or less the same scale so the effect of economies of scale is less relevant. As most costs are incurred in the same year inflation or discounting were less relevant. Results will be the same as all systems use similar numbers of consumables in the same time periods.

### Future research

Surveillance for other diseases could possibly be done on samples of eggs. Current systems in the Dutch egg layer sector use blood (MG and ND), manure or environmental (Salmonella) samples. At present no epidemiological studies are available that compare the effectiveness of the current programs with programs based on egg samples. Improvements in animal welfare, higher surveillance effectiveness and lower surveillance costs seem to be possible, if more surveillance would be done on egg samples.

The current economic model can be used to calculate the costs of surveillance programmes with a different aim from the programme here studied. For example a similar study could be done on the costs of surveillance for salmonella, ND and Mg based on egg yolk samples. This economic model could also be of interest for an early warning programme that allows rapid detection of LPAI virus introduction in laying hens, and therefore reduces the probability of both spread of the LPAI viruses to other farms and mutation of the LPAI viruses to HPAI viruses. Such surveillance programme would require higher sampling frequency and sampling size than the programme studied here and this model can be used to evaluate the economic impact of increased sampling either of blood or egg samples.

## Supporting Information

Appendix S1 **Evaluation of the effectiveness of the surveillance systems.**
(DOC)

Table S1 **Input variables of the model, including values, units and data sources.**
(DOC)
