# Supplementary material for: Cost Analysis of Various Low Pathogenic Avian Influenza Surveillance Systems in the Dutch Egg Layer Sector
Source: PLoS One. 2012 Apr 16;7(4):e33930. doi: 10.1371/journal.pone.0033930 (PMC3327686; doi:10.1371/journal.pone.0033930)
Supplement: Appendix S1 — Evaluation of the effectiveness of the surveillance systems. (DOC) [file pone.0033930.s001.doc]

**Appendix S1. Evaluation of the effectiveness of the surveillance systems.**

The effectiveness of a surveillance system was evaluated by quantifying its sensitivity. The sensitivity of a surveillance system (here referred to as Surveillance Sensitivity (SSe)) carried out in egg layer farms in the Netherlands using blood or egg samples was estimated using a scenario tree model .

The effectiveness to test an individual infected farm positive for LPAI is estimated (here referred to as Flock Sensitivity ($F_{se}$) by:

| $F_{se}=1-(1-f^{*}\bullet e\bullet\theta)^{m}$ | (1) |
| --- | --- |

where $f^{*}$ is the design prevalence , $e$is the expected egg production of infected chickens (only applies when eggs are used as samples), $\theta$is the sensitivity of the commercial ELISA test used to test blood or egg samples, and $m$is the number of blood (30) or egg (35) samples taken from each farm. Table S1 describes the values and distributions given to these parameters. Note that for this evaluation $f^{*}$= 10% is used which is lower (more sensitive) than the design prevalence of 30% recommended by the European Union .

To estimate the $SSe$, two components were considered: 1) component $I$, which represents the surveillance carried out in indoor layer farms and 2) component$O$, which represents the surveillance carried out in outdoor layer farms. First the sensitivity of surveillance was estimated in each of these components, which is called Component Sensitivity ($CSe$) and is given by $CSe_{j}=1-(1-P_{fj})^{n}$. Where$P_{fj}$is the probability that an indoor ($P_{fI}$) or outdoor ($P_{fO}$) farm is infected, and$n$is the number of farms (indoor or outdoor) sampled. For indoor farms which are sampled once a year, $P_{fI}$is given by:

| $P_{fI}=i\bullet p^{*}\bullet F_{se}$ | (2) |
| --- | --- |

Where $i$ is the proportion of indoor farms in the Netherlands, $p^{*}$the farm level design prevalence and $F_{se}$ the flock sensitivity. For outdoor farms, which are sampled four times per year, $P_{_{fO}}$is given by the sum of the probabilities of a farm being infected and detected in the first sampling or in the second, third or fourth sampling conditional on escaping detection (classified as negative) in the previous samplings:

| $P_{fO}=o\bullet p^{*}\bullet F_{se}\sum_{k=o}^{3} (1-F_{se})^{k}$ | (3) |
| --- | --- |

In equation (3), $o$is the proportion of outdoor farms in the Netherlands. Note that for this evaluation, $p^{*}$=0.05% was used, which is lower than the 5% recommended by the European Union .

Finally, the $SSe$ was estimated, which is the complement of the probability that all indoor and outdoor farms are classified as non-infected. This $SSe$ (of a system sampling blood or of a system sampling eggs) is given by:

| $SSe=1-((1-CSe_{I})(1-CSe_{O}))$ | (4) |
| --- | --- |

Models (for systems using eggs or blood) were implemented in Excel® using the add-in software Poptools . Stochastic simulations of 500 iterations for each model were performed.

Table S2: Values of the variables used in the Scenario Tree model to estimate the Flock and Component sensitivity for LPAI. Values are either a single point estimate or a distribution.

| Variable | Value | Description | Reference |
| --- | --- | --- | --- |
| *Flock Sensitivity* $F_{se}$ | | | |
| $f^{*}$ | 10 % | Within flock design prevalence |  |
| $e$ | Pert(0.72,0.79,0.83)^a^ | Probability of an infected chicken producing an egg |  |
| $\theta$ | Pert(0.96,0.99,1.00)^a^ | Sensitivity of the commercial ELISA test. |  |
| $m$ | Blood = 30; eggs = 35 | Sample size |  |
| *Component Sensitivity* $CSe$ | | | |
| $p^{*}$ | 0.05% | Farm level design prevalence |  |
| $n$ | Indoor farms = 838 | Number of farms in the Netherlands. All farms are sampled. |  |
|  | Outdoor farms = 270 |  |
| $i$ | 0.76 | Proportion of indoor layer farms in The Netherlands. |  |
| $o$ | 0.24 | Proportion of outdoor layer farms in The Netherlands |  |

^a^ Pert Distribution (a, b, c) where a = the minimum, b = the most likely and c = the maximum value.

References

1. Martin PAJ, Cameron AR, Greiner M (2007) Demonstrating freedom from disease using multiple complex data sources: 1: A new methodology based on scenario trees. Prev Vet Med 79: 71-97.

2. European Commission (2007) Commission Decision 2007/268/EC of 13 April 2007 on the implementation of surveillance programmes for avian influenza in poultry and wild birds to be carried out in the Member States and amending Decision 2004/450/EC. OJEU L 115: 3.5.2007, p.2003.

3. Gonzales JL, Elbers ARW, Bouma A, Koch G, Wit JJd, et al. (2010) Low-pathogenic notifiable avian influenza serosurveillance and the risk of infection in poultry - a critical review of the European Union active surveillance programme (2005 - 2007). Influenza and Other Respiratory Viruses 4: 91-99.

4. Hood GM (2010) PopTools version 3.2.3.

5. Gonzales JL, Elbers ARW, Bouma A, Koch G, de Wit JJ, et al. (In Press) Transmission characteristics of low pathogenic avian influenza virus of H7N7 and H5N7 subtypes in layer chickens. Veterinary Microbiology (2011), doi:10.1016/j.vetmic.2011.09.016.

6. Jeong O-M, Kim M-C, Kang H-M, Ha G-W, Oh J-S, et al. (2010) Validation of egg yolk antibody based C-ELISA for avian influenza surveillance in breeder duck. Veterinary Microbiology 144: 287-292.

7. Beck JR, Swayn DE, Davison S, Casavant S, Gutierrez C (2003) Validation of egg yolk antibody testing as a method to determine influenza status in white leghorn hens. Avian Diseases 47: 1196-1199.

8. Anonymous (2010) Pluimveevlees en Eieren, Statistisch jaarrapport het jaar 2009. Zoetermeer: Product Boards for Livestock, Meat and Eggs 4: 25 - 38 p.
