# Supplementary material for: Cost Analysis of Various Low Pathogenic Avian Influenza Surveillance Systems in the Dutch Egg Layer Sector
Source: PLoS One. 2012 Apr 16;7(4):e33930. doi: 10.1371/journal.pone.0033930 (PMC3327686; doi:10.1371/journal.pone.0033930)
Supplement: Table S1 — Input variables of the model, including values, units and data sources. (DOC) [file pone.0033930.s002.doc]

*Table S1: Input variables of the model, including values, units and data sources.*

| **Variable** | **Description** | **Value** | **Unit** | **Explanation** | | **Source** |
| --- | --- | --- | --- | --- | --- | --- |
| **Sampling** |  |  |  |  | |  |
| NF_in_ | Number of egg laying farms (indoor) | 838 | # |  | |  |
| NF_out_ | Number of egg laying farms (outdoor) | 270 | # |  | |  |
|  | Number of egg laying farms delivering to large packing station | 475 |  |  | |  |
| NSY_in_ | Number of samplings per year per farm (indoor) | 1 | #/year/farm |  | |  |
| NSY_out_ | Number of samplings per year per farm (outdoor) | 4 | #/year/farm |  | |  |
| NSF_blood_ | Number of blood samples per farm | 30 | #/farm/sampling |  | |  |
| NSF _egg_ | Number of egg samples per farms | 35 | #/farm/sampling |  | |  |
| EV | Egg value | 0.0556 | €/egg | Average egg price over the year 2005 - 2009 | |  |
| CCV | Call out charge veterinarian | 20.58 | €/visit |  | |  |
| LC_vet_ | Hourly rate veterinarian | 116.17 | €/hour |  | |  |
| Hours_bs_ | Hours for preparation and blood sampling | 0.667 | hour |  | |  |
|  |  |  |  |  | |  |
| SCS_in_ | Share cost attributed to AI surveillance (indoor farms) | 33.3 | % | Sampling for three diseases so one third for Avian influenza | | Calculation |
| SCS_out_ | Share cost attributed to AI surveillance (outdoor farms) | 83.3 | % | Of four samplings one combined sampling of which one third is taken. (Note that only sampling is combined and separate tests are used.) | | Calculation |
|  |  |  |  |  | |  |
| hours_F_ | Hours needed per sampling (farm) | 0.333 | h/sample |  | | Best guess |
| hours_PS_ | Hours needed per sampling (PS) | 0.167 | h/sample |  | | Best guess |
| Lc_low_ | The labour cost of a worker | 12.06 | €/hr | Based on €25,000 a year and 1983.6 working hours a year | |  |
| PM_F_ | Packing material (farm) | 1 | € | A cardboard box with protective material | | Best guess |
| PM_PS_ | Packing material (PS) | 0.25 | € | It is estimated that 4 batches of eggs can be send in one package for the price of sending one package. | | Best guess |
| **Sample preparation** | |  |  |  | |  |
| SH_blood_ | Number of blood samples per hour prepared by a robot | 330 |  | | Sample/hour | |
| PPR_blood_ | Price robot blood sample preparation | 50000 | € |  | |  |
| EVR_blood_ | End value robot blood sample preparation | 0 | € |  | |  |
| TLT_blood_ | Technical life time robot blood sample preparation | 5 | years |  | |  |
| MY_blood_ | Maintenance costs robot blood sample preparation | 1604 | €/year |  | |  |
| MT_blood_ | Material cost robot blood sample preparation | 0.1 | €/test | Assumed to be the same percentage (3.2%) of the purchase price as it is for an egg-robot. | |  |
| OC_blood_ | Operating costs | 0.061107 | €/test |  | |  |
| WYD | Number of working days per year | 252 | Days/year |  | | Best guess |
| HWD | Number of working hours per day | 8 | Hours/day |  | | Best guess |
| SH_egg_ | Number of egg samples per hour prepared by a robot | 165 | Samples/hour |  | |  |
| PPR_egg_ | Price robot egg sample preparation | 105000 | € |  | |  |
| EVR_egg_ | End value robot egg sample preparation | 0 | € |  | |  |
| TLT_egg_ | Technical life time robot egg sample preparation | 5 | years |  | |  |
| MY_egg_ | Maintenance robot egg sample preparation | 3370 | €/year |  | |  |
| MT_egg_ | Material costs robot egg sample preparation | 0.1 | €/test |  | |  |
| OC_egg_ | Operating costs | 0.122214 | €/test | Based on the number test samples that can be prepared per day and assuming 8 working hours (7 operating hours and 1 for cleaning) with a labour cost of € 35,000for 1983.6 working hours a year. | |  |
| CSS | Spinning down and booking in blood samples | 0.027132 | €/sample | Based on 2 minutes for booking in a batch of 30 samples, 1.5 minutes to load 120 samples in a centrifuge and 6 minutes for centrifuging with a labour cost of € 35,000 for 1983.6 working hours a year. | |  |
| BS | Booking in egg samples | 0.014004 | €/sample |  | |  |
| NR_E/P/P_, | Number of robot, each packing station a robot. | 8 | # |  | |  |
| NR_LP_ | Number of robots on large packing stations | 4 | # | Number of large packing stations based on data provided by the product board, packing station with at least 75 farms contracted. | |  |
| **Transport** |  |  |  |  | |  |
| PPP | Price package <10 kg (postal service) | 6.75 | €/package |  | |  |
| DPL | Totally travelled distance to central lab (25 largest packing stations) | 3952 | Km | Shortest route calculated using google maps. Based on the number and type of farms the number of transports is calculated using product board data and the robot capacity | |  |
| CTK | Cost of transport per km | 0.490056 | €/km | Based on € 0.28 /km for a van and € 0.19 /km for a driver with an average driving speed of 60 km/h and a salary of € 25,000 for 1983.6 working hours a year. | |  |
| **ELISA test** | |  |  |  | |  |
| PT | Price test | 3.36 | €/sample | The commercial price is decreased with the price for the preparation of 1 blood sample = ($\frac{SPB}{NFB_{Blood/Farm/Lab}\bullet(NSY_{indoor}+NSY_{outdoor})}$) | |  |
| **Waste** |  |  |  |  | |  |
| AWB | Amount of waste from blood sample preparation | 0.035 | kg/test |  | | Best guess |
| AWE | Amount of waste from egg sample preparation | 0.075 | kg/test |  | | Best guess |
| PDW | Price of waste destruction | 2 | €/kg | Based on € 5 per full barrel of waste and € 1.84 per kg of filling under the assumption of 30 kg of waste per barrel. | |  |
| **Communication** | |  |  |  | |  |
| PNM | Price sending message | 0.5 | €/farms tested | € 0.44 for postal service + €0.06 for processing at lab | | Best guess, |
| **Positive results** | |  |  |  | |  |
| PFP | Percentage of positive | 2 | % |  | |  |
| TPS_blood_ | Transport of blood samples for confirmation test | 521 | €/year | Using the robot capacity, the number of samples and the percentage of false positives the lab would have 7 samples a day on average. Therefore it is assumed that the samples are transported in batches of 7 | | Google maps |
| TPS_egg_ | Transport of egg samples for confirmation test | 608 | €/year |  | |  |
| PCT | Price of confirmation test | 10 | €/sample |  | |  |

**References**

1. Anonymous (2010) Pluimveevlees en Eieren, Statistisch jaarrapport het jaar 2009. Zoetermeer: Productboards for Livestock, Meat and Eggs. 4, 25 - 38 p.

2. Personal communication PPaE (2011) Sector expert of Productboard Poultry and Eggs (PPE). Zoetermeer.

3. Elbers ARW, de Wit JJ, Hulsbergen HBA, van der Spek AN, Fabri THF, et al. Avian influenza surveillance in poultry in the Netherlands between 2004-2006.; 2007 10 - 15 September 2007; Beijing, China.

4. Gonzales JL, van der Goot JA, Stegeman JA, Elbers ARW, Koch G (2011) Transmission between chickens of an H7N1 Low Pathogenic Avian Influenza virus isolated during the epidemic of 1999 in Italy. Veterinary Microbiology 152: 187-190.

5. Velthuis AGJ, Mourits MCM, Saatkamp HW, de Koeijer AA, Elbers ARW (2011) Financial Evaluation of Different Vaccination Strategies for Controlling the Bluetongue Virus Serotype 8 Epidemic in the Netherlands in 2008. Plos One 6.

6. Personal communication Pv (2011) Veterinarian specialized in the poultry sector. Wageningen.

7. Livestock Research WU (2009) Quantitative Information Livestock (KWIN-V). Lelystad: Wageningen UR Livestock Research.

8. Personal communication AHS (2011) Veterinary expert of Animal Health Service (GD Deventer). Deventer.

9. PostNL (2011) Dutch national postal service PostNL. Utrecht.

10. Anonymous (2008) Survey programme for Avian Influenza in poulty and wild birds, the Netherlands. In: general HCd-, editor. Brussel.
